# The Engineering of a Novel Ligand in gH Confers to HSV an Expanded Tropism Independent of gD Activation by Its Receptors

**DOI:** 10.1371/journal.ppat.1004907

**Published:** 2015-05-21

**Authors:** Valentina Gatta, Biljana Petrovic, Gabriella Campadelli-Fiume

**Affiliations:** Department of Experimental, Diagnostic and Specialty Medicine, University of Bologna, Bologna, Italy; University of California, Irvine, UNITED STATES

## Abstract

Herpes simplex virus (HSV) enters cells by means of four essential glycoproteins - gD, gH/gL, gB, activated in a cascade fashion by gD binding to one of its receptors, nectin1 and HVEM. We report that the engineering in gH of a heterologous ligand – a single-chain antibody (scFv) to the cancer-specific HER2 receptor – expands the HSV tropism to cells which express HER2 as the sole receptor. The significance of this finding is twofold. It impacts on our understanding of HSV entry mechanism and the design of retargeted oncolytic-HSVs. Specifically, entry of the recombinant viruses carrying the scFv-HER2–gH chimera into HER2^+^ cells occurred in the absence of gD receptors, or upon deletion of key residues in gD that constitute the nectin1/HVEM binding sites. In essence, the scFv in gH substituted for gD-mediated activation and rendered a functional gD non-essential for entry *via* HER2. The activation of the gH moiety in the chimera was carried out by the scFv *in cis*, not *in trans* as it occurs with wt-gD. With respect to the design of oncolytic-HSVs, previous retargeting strategies were based exclusively on insertion in gD of ligands to cancer-specific receptors. The current findings show that (i) gH accepts a heterologous ligand. The viruses retargeted *via* gH (ii) do not require the gD-dependent activation, and (iii) replicate and kill cells at high efficiency. Thus, gH represents an additional tool for the design of fully-virulent oncolytic-HSVs retargeted to cancer receptors and detargeted from gD receptors.

## Author Summary

To enter cells, all herpesviruses use the core fusion glycoproteins gH/gL and gB, in addition to species-specific glycoproteins responsible for specific tropism, etc. In HSV, the additional glycoprotein is the essential gD. We engineered in gH a heterologous ligand to the HER2 cancer receptor. The recombinant viruses entered cells through HER2, independently of gD activation by its receptors, or despite deletion of key residues that are part of the receptors’ binding sites in gD. The ligand activated gH *in cis*. Cumulatively, the receptor-binding and activating functions of gD were no longer essential and were replaced by the heterologous ligand in gH. Relevance to translational medicine rests in the fact that gH can serve as a tool to retarget HSV tropism to cancer-specific receptors. This expands the toolkit for the design of fully-virulent oncolytic-HSVs.

## Introduction

Entry of herpes simplex virus (HSV) into the cell is a multistep process that involves four virion glycoproteins (gD, gH/gL, gB), all of which are required. gD is species-specific, and a major determinant of HSV tropism. gH/gL and gB constitute the conserved fusion apparatus across the *Herpesviridae* family; gB exhibits features typical of viral fusion glycoproteins [1–6]. Many steps in the HSV entry process remain to be elucidated and the overall model is partly speculative. Inasmuch as the process initiates with gD binding to one of its receptors, and culminates with gB-mediated virion-cell fusion, the commonly accepted model envisions that the four viral glycoproteins are activated in a cascade fashion by the receptor-bound gD through intermolecular signaling among the glycoproteins themselves [1]. Specifically, following virion attachment to cells, the interaction of gD with one of its alternative receptors—nectin1, HVEM, and modified heparan sulphates [7–10]—results in conformational modifications to gD, in particular in the dislodgement of the ectodomain C-terminus, which carries the profusion domain [11–15]. Since this domain can interact with the heterodimer gH/gL [16,17], most likely this step is a critical event in the activation cascade. Recently, we have shown that gH/gL interacts with two interchangeable receptors, αvβ6- and αvβ8-integrins, which promote HSV endocytosis, and most likely participate in the process of gH/gL activation [18]. Evidence for the activation cascade and for intermolecular signaling among the glycoproteins is indirect and rests on three sets of data: interactions among the four glycoproteins [17,19,20]; the ability of soluble gD to rescue the infection of gD^-/-^ non-infectious virions and to promote fusion in a cell-cell fusion assay; the ability of soluble gD receptor to mediate virus entry into receptor-negative cells [15,21–23].

There is intense interest in HSV as an oncolytic agent (o-HSV) [24–27]. In the first and second generations o-HSVs, now in clinical trials, safety was obtained at the expense of virulence through single or multiple deletions. The most successful example is T-VEC, a HSV recombinant deleted in both copies of the γ_1_34.5 gene and of ICP47 gene, and encoding the GM-CSF cytokine to boost the host immune response against the tumor [28]. In a phase III clinical trial, T-VEC improved the outcome of patients carrying metastatic melanoma [29]. A drawback of attenuation is that it strongly reduces the range of tumors against which the o-HSVs are effective. Thus, deletion of the γ_1_34.5 genes restricts o-HSVs replication to cells defective in the PKR-dependent innate response. To overcome these limitations, non-attenuated o-HSVs retargeted to cancer-specific receptors and detargeted from the natural receptors were designed. They preserve the killing ability of wt-viruses [30,31]. So far, retargeting strategies entailed genetic modifications to gD, in particular the insertion of novel ligands, coupled with appropriate deletions for detargeting purposes [30,32–38]. The heterologous ligands included the IL13 cytokine, urokinase-type plasminogen activator or single chain antibodies (scFvs). The retargeting through genetic modifications obtained in the above-mentioned studies has clear advantages over retargeting through coupling of appropriate moieties to virions, and even more so over non-replicating viruses (see, for example [39]). In the former case virions maintain the retargeted phenotype generation after generation, even during replication in the tumor. In the latter case, targeting occurs only for a single generation, and viruses are usually non-detargeted, hence they also infect non cancer cells. Furthermore, non-replicating virions fail to propagate the therapeutic effect beyond the initially infected tumor cells.

The cancer-specific receptor selected in our laboratory is HER2 (human epidermal growth factor receptor 2), a member of the EGFR (epidermal growth factor receptor) family, overexpressed in breast, ovary, gastric carcinomas, glioblastomas, etc [40]. Two fully retargeted o-HSVs were generated. They differ in the portions of gD that were deleted for detargeting purposes. R-LM113 carries the deletion of the AA 6–38 N-terminal region [32,33]. R-LM249 carries the deletion of the 61–218 core region [34]. In both viruses, the deleted sequences were replaced with the scFv to HER2 derived from trastuzumab, a humanized MAb now in clinical practice. The scFv binds HER2 at high affinity (29.3 nM) [41]. In preclinical studies R-LM113 and R-LM249 exerted therapeutic effects against human breast and ovary cancers, and against a murine model of HER2^+^ glioblastoma [32–35,42]. Intraperitoneally-administered R-LM249 exerted therapeutic effect against metastases of ovary and breast cancers diffuse to the peritoneum, or to the brain [35].

Here, we engineered a heterologous ligand in gH. The aims were twofold, i.e. to better elucidate the respective roles of gD and gH/gL in HSV entry, and define whether gD is an absolute requirement for HSV entry, and to explore novel avenues in the design of retargeted o-HSVs. We report that the engineering in gH of a scFv to HER2 confers to the recombinant viruses the ability to use HER2 as the sole receptor, in the absence of gD receptors, or upon deletion of residues that form the nectin1/HVEM binding sites in gD.

## Results

### Design of R-VG803 and R-VG809, and verification of chimeric gH

**Fig 1:**
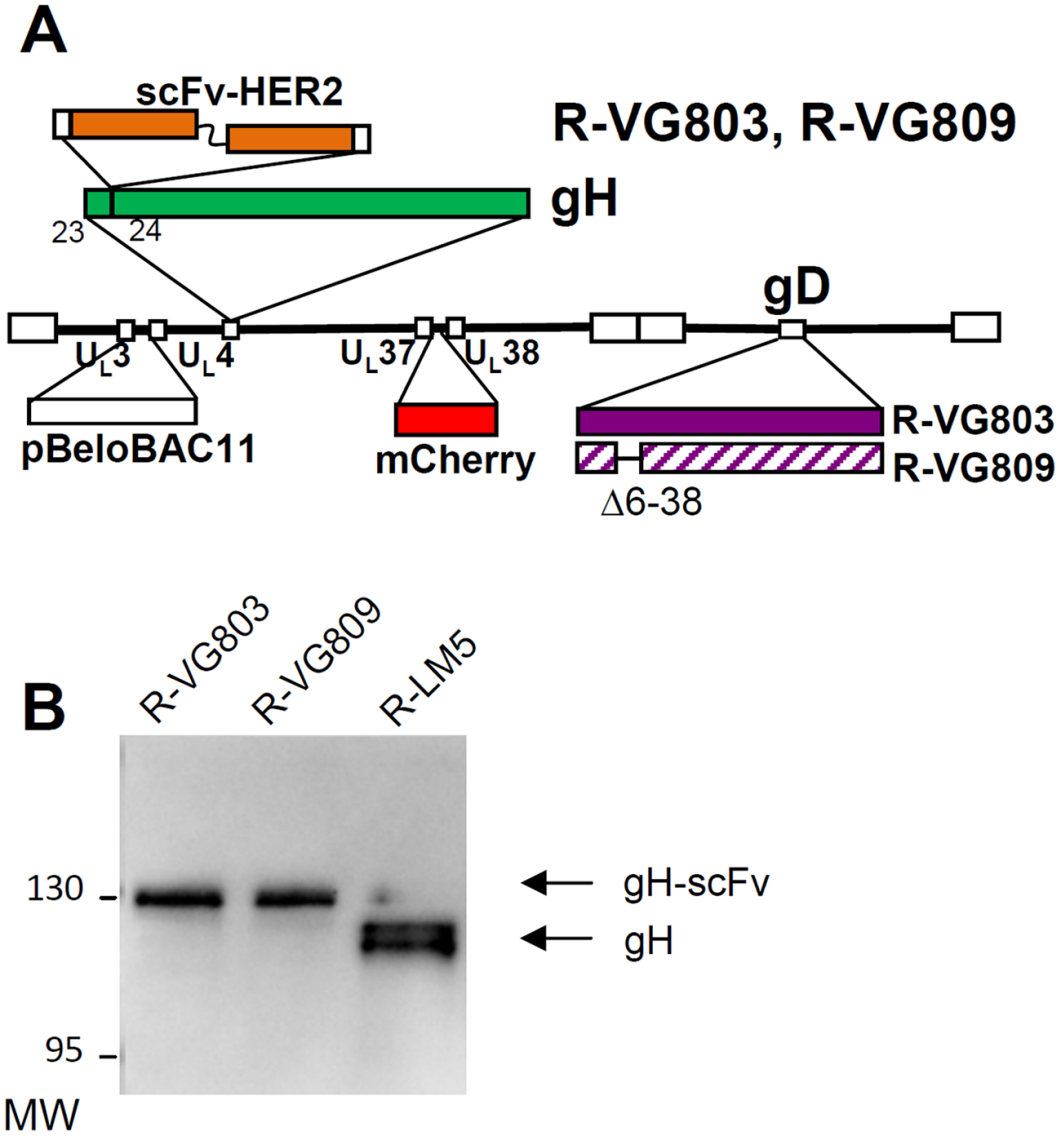
(A) Schematic drawing of R-VG803 and R-VG809 genomes. Sequence arrangement of HSV-1 genome shows the inverted repeat sequences as rectangular boxes. The scFv-HER2 sequence (VL-linker-VH) is inserted between AA 23 and 24 of gH, bracketed by upstream and downstream Gly-Ser linkers, 8 and 12 AA long, respectively. LOX-P-bracketed p-Belo-BAC and mCherry sequences are inserted between UL3-UL4, and between UL37-UL38 regions, respectively. The gD sequence is wt in R-VG803, and carries the deletion of the AA 6–38 region in R-VG809. (B) R-VG803 and R-VG809 express the chimeric scFv—gH glycoprotein. Lysates of Vero cells infected with R-VG803, R-VG809 or R-LM5 were subjected to PAGE. gH was detected by immunoblot [44]. Figures on the left represent the migration position of the 130K and 95K MW markers.

R-VG803 carries the insertion of the scFv to HER2 (herein named scFv-HER2) at the N-terminus of gH, the mCherry red fluorescent marker in the UL37–UL38 intergenic region, and the LoxP-bracketed BAC sequences between UL3 and UL4 (schematic representation in Fig 1A). R-VG809 carries the deletion of the AA 6–38 portion in gD, and is otherwise identical to R-VG803. The recombinant viruses were generated by transfection of the recombinant BAC-genomes into SK-OV-3 cells, a HER2^+^ cell line derived from human ovary carcinoma, and resistant to trastuzumab [35]. The presence of the scFv—gH chimera in R-VG803 and R-VG809 was verified by sequencing of the entire ORF, and by immunoblot of Vero cells infected with R-VG803, R-VG809, or R-LM5 [43]. The latter is essentially a wt-virus with genetic modifications similar to those of R-VG803 and R-GV809, i.e. it carries wt-gD, the LoxP-bracketed BAC sequences, and EGFP (Enhanced green fluorescence protein) instead of mCherry. The annotated scFv-gH sequence is reported in S1 Fig. For immunoblotting, infected cell lysates were subjected to SDS-PAGE (sodium dodecyl sulphate polyacrylamide gel electrophoresis), and the blots were immunoreacted to polyclonal antibody (PAb) to gH [44]. The chimeric scFv—gH migrated with a slower electrophoretic mobility than wt-gH from R-LM5, and an apparent M_r_ of 130 K (Fig 1B).

### R-VG803 infects cells that express HER2 as the sole receptor, in the absence of a gD receptor

**Fig 2:**
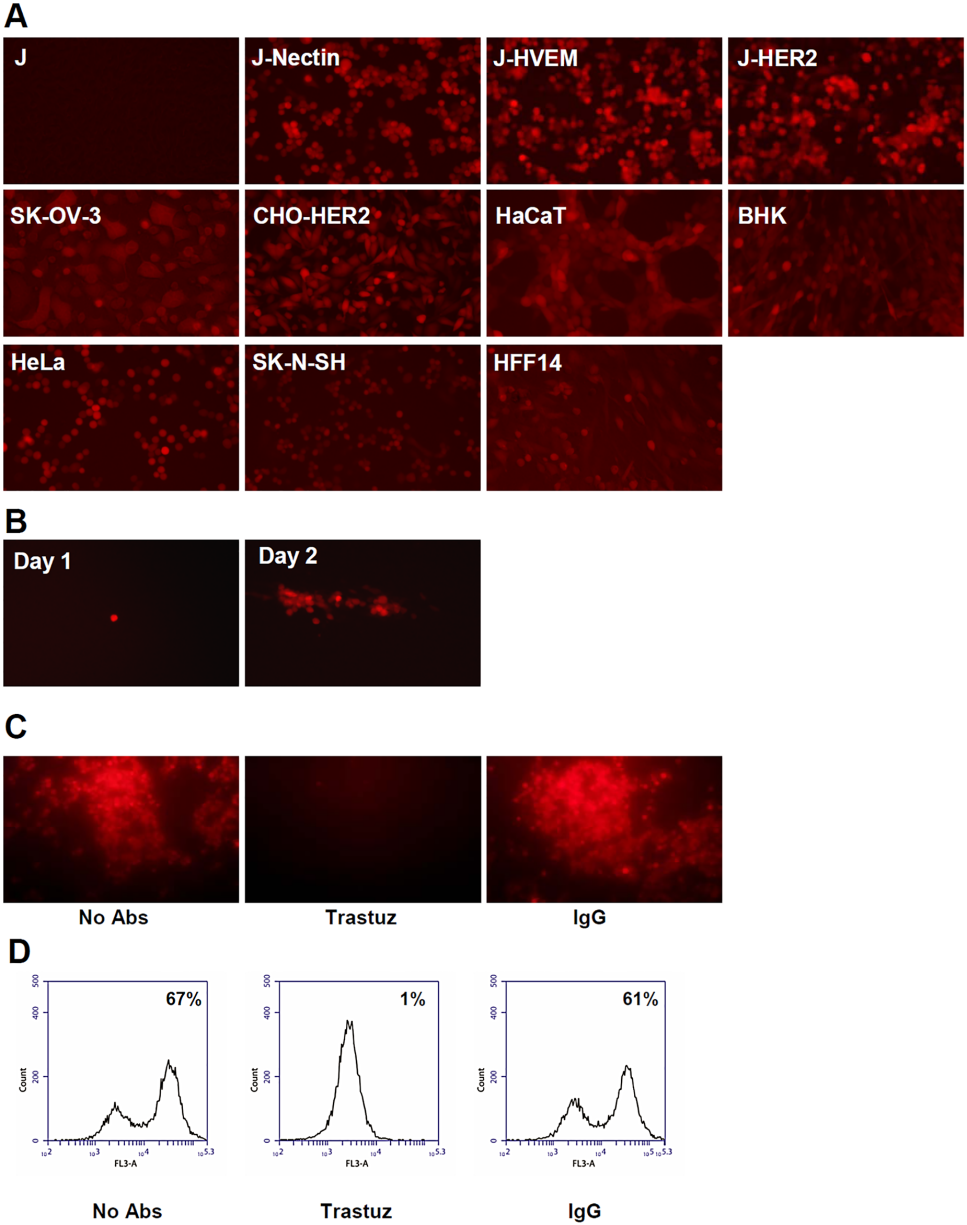
R-VG803 infects cells that express HER2 as the sole receptor (J-HER2 cells) in a HER2-dependent manner, and progeny virus spreads in these cells. J cells express no receptor for wt-HSV. J-HER2, J-Nectin, J-HVEM only express the indicated receptor. (A) The indicated cells were infected with R-VG803 (2 PFU/cell as titrated in SK-OV-3), and monitored for red fluorescence microscopy. (B) J-HER2 cells were infected with R-VG803 (0.01 PFU/cell), overlaid with medium containing the neutralizing MAb 52S (ascites fluid 1:10,000) [45], and monitored daily for red fluorescence. Pictures of a same plaque are shown. (C, D) Trastuzumab inhibits R-VG803 infection of J-HER2 cells. J-HER2 cells were infected with R-VG803 in the presence of trastuzumab (trastuz) (28 μg/ml, final concentration) or control IgGs (28 μg/ml, final concentration). Infection was monitored by fluorescence microscopy (C), or flow cytometry (D). All pictures in panel A were taken with an exposure time of 0.6 sec. The whole pictures showing SK-OV-3- and HFF14-infected cells were adjusted as follows; increase in brightness 25%, increase in contrast 50%. Panel B pictures were adjusted as follows, increase in brightness 20%, increase in contrast 30%.

Initially, we engineered R-VG803. To test whether it can use HER2 as an entry receptor, we made use of J-HER2 cells. The parental J cells express no receptor for gD, hence cannot activate gD, and are not infected by wt-HSV [7]. J-HER2 cells transgenically express HER2 as the sole receptor [43]. As controls, we included J-nectin and J-HVEM cells, which transgenically express nectin1 or HVEM as receptors and are infected by wt-HSV [7], and a panel of human and animal cells, which express the human or animal nectin1/HVEM. The panel included CHO, BHK, keratinocytic HaCaT, human fibroblastic HFF14, epithelial HeLa, the neuronal SK-N-SH cells, and the HER2-positive SK-OV-3 cancer cells. As shown in Fig 2A, R-VG803 infected J-HER2 cells. The infection of J-nectin1, J-HVEM, and of the animal and human cells (Fig 2A) was not surprising, given that R-VG803 encodes a wt-gD. Furthermore, R-VG803 could perform cell-to-cell spread in J-HER2 cells. Cells were infected at 0.01 PFU/cell, overlaid with medium containing MAb 52S (ascites fluid 1:10,000). At day 1 infection involved single cells. In the following day infection involved clusters of cells (Fig 2B).

We confirmed that R-VG803 infection occurs through the HER2 receptor, by blocking the infection with trastuzumab, in fluorescence microscopy (Fig 2C), and flow cytometry (Fig 2D) assays. The results validate the inference that R-VG803 uses HER2 as the portal of entry in J-HER2 cells. This finding supports two fundamental conclusions. First, infection with a gH-retargeted HSV can take place in the absence of a gD receptor. Under these conditions, gD is physically present but functionally ablated as receptor-binding glycoprotein, as it can not be activated by its cognate receptor(s) and can not transmit the activation to gH. Second, the tropism of HSV can be modified by engineering a heterologous ligand in gH.

### Receptor usage in cells that harbour both HER2 and nectin1/HVEM

**Fig 3:**
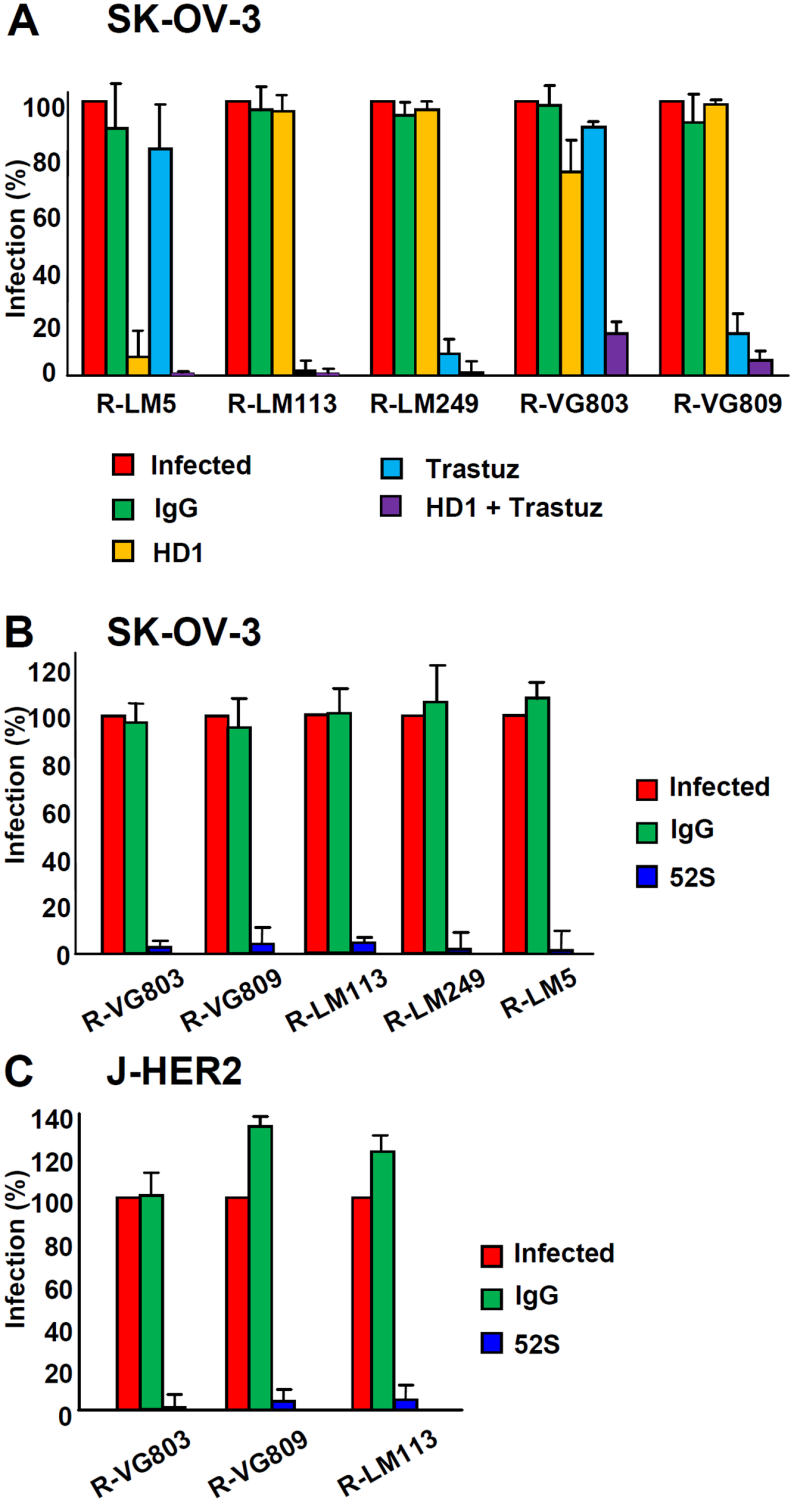
Receptor usage by R-VG803 and R-VG809 in SK-OV-3 cells, detected through inhibition of infection by trastuzumab, MAb HD1, or combination thereof. The indicated viruses were preincubated with HD1 (1.5 μg IgG/ml, final concentration) and then allowed to infect SK-OV-3 cells. When indicated, cells were pretreated with trastuzumab (28 μg/ml, final concentration), or control IgGs. Extent of infection was quantified 24 h later by means of flow cytometry, and expressed as percentage relative to cells infected with untreated virus, or untreated cells. Each value represents the average of three independent experiments ± S.D. (B, C) R-VG803 and R-VG809 infection of SK-OV-3 (B) and J-HER2 (C) cells is inhibited by MAb 52S to gH. The indicated virions were preincubated with MAb 52S (ascites fluid 1:25), or mouse IgGs for 1 h, prior to infection of SK-OV-3 or J-HER2 cells (2 or 0.3 PFU/cell, respectively), until harvesting at 24 h after infection. Infection was quantified by flow cytometry and expressed as % of cells infected with untreated virions. Each value represents the average of three independent experiments ± S.D.

We analysed the receptor usage in cells that express both sets of receptors, HER2 and nectin1/HVEM, exemplified by SK-OV-3 cells. The question was whether one receptor was preferentially used over the other, or each one was used alternatively. In the latter case, we expected that a block in the access to one of the two sets of receptors—e.g. HER2—should result in low extent of inhibition, whereas the simultaneous block to both sets of receptors should result in strong inhibition. The latter was indeed the case. As controls, we included the two retargeted viruses R-LM113 and R-LM249, and wt R-LM5. R-LM113 is detargeted from natural gD receptors [33,43], even though the AA 6–38 deletion in gD removes only some residues implicated in the nectin1-binding site, in addition to the entire HVEM binding site. The nectin1 binding site is widespread in the molecule, and includes the Ig-folded core and portions located between AA 35–38, 199–201, 214–217, 219–221 [12,13,34]. SK-OV-3 cells were infected with the indicated viruses, in the presence of trastuzumab, MAb HD1 to gD, or both. Fig 3A shows that trastuzumab or HD1 exerted almost no inhibition on R-VG803 when given singly, but practically abolished infection when given together. In contrast, R-LM113 and R-LM249 were inhibited by trastuzumab alone. Thus, R-VG803 can use alternatively HER2 or nectin1/HVEM to infect SK-OV-3 cells. Usage of one or the other portals of entry by R-VG803 depends on the spectrum of receptors displayed by the cells.

To characterize further the scFv—gH chimera we asked whether infection can be blocked by the neutralizing MAb 52S to gH. This MAb recognizes a continuous epitope, independent of gL, with critical residues at S536 and A537 [45,46]. R-VG803 infection of both SK-OV-3 and J-HER2 cells was abolished by MAb 52S (ascites fluid 1:25) (Fig 3B and 3C), indicating that a key functional domain in wt-gH was preserved in the chimera.

### Deletion of AA 6–38 from R-VG803 gD results in a recombinant retargeted to HER2 *via* gH and detargeted from gD receptors

**Fig 4:**
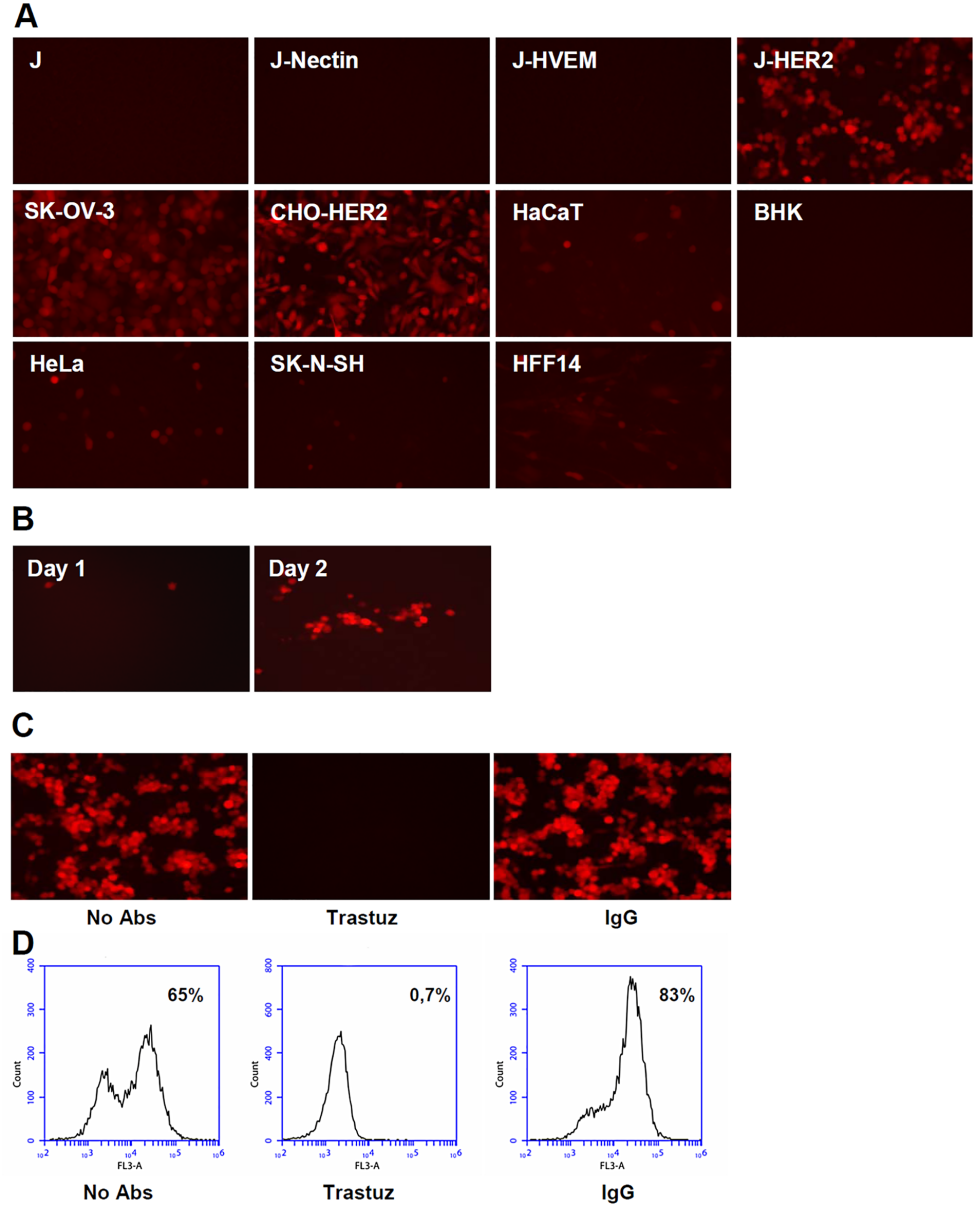
R-VG809 infects cells that express HER2, fails to infect cells *via* gD receptors, and progeny virus spreads in J-HER2 cells. (A) The indicated cells were infected with R-VG809 (20 PFU/cell as titrated in SK-OV-3), and monitored for red fluorescence microscopy at 24 h after infection. (B) J-HER2 cells were infected with R-VG809 (0.01 PFU/cell), overlaid with medium containing the neutralizing MAb 52S to gH (ascites fluid, 1:10,000), and monitored daily for red fluorescence. Daily pictures of a same plaque are shown. (C, D) Trastuzumab inhibits R-VG809 infection of J-HER2 cells. J-HER2 cells were infected with R-VG809 in the presence of trastuzumab (trastuz) (28 μg/ml, final concentration) or control IgGs (28 μg/ml, final concentration). Infection was monitored by fluorescence microscopy (C), or flow cytometry (D). Panel B pictures were adjusted as follows, increase in brightness 20%, increase in contrast 30%.

Inasmuch as R-VG803 infects J-HER2 cells independently of gD receptors and of neutralizing MAb to gD, we reasoned that it might be possible to engineer a recombinant carrying the scFv-HER2 in gH and the deletion of receptors’ binding sites from gD. We deleted the AA 6–38 region. R-VG809 failed to infect not only J-HVEM cells, but also J-nectin cells, and did not infect or infected very little the panel of animal and human cell lines employed above. It infected efficiently J-HER2, CHO-HER2 and SK-OV-3 cells (Fig 4A). In summary, R-VG809 exhibited a redirected tropism, strikingly different from that of R-VG803 (compare Fig 4A with Fig 2A). R-VG809 was also capable of cell-to-cell spread in J-HER2 cells (Fig 4B). Further validation that R-VG809 uses HER2 as portal of entry was provided by inhibition with trastuzumab (Fig 4C and 4D).

Analysis of the inhibitory effect of trastuzumab and MAb HD1 in SK-OV-3 cells shows that R-VG809 infection was inhibited by trastuzumab, even in the absence of MAb HD1 (Fig 3A), confirming that HER2 is the only portal for R-VG809. Infection of R-VG809 was blocked by MAb 52S, in agreement with the fact that R-VG809 and R-VG803 carry the same gH chimera (Fig 3B and 3C). We conclude that R-VG809 infection *via* the HER2-retargeted gH does not require the receptors’ binding sites in gD, and the receptor-mediated gD activation. Inasmuch R-VG809 does not carry the deletion of the entire gD open reading frame, we cannot formally rule out that gD serves a hypothetical, additional, so-to-say structural function, i.e. it facilitates the formation and/or stabilization of complexes among the glycoproteins.

### Characterization of the gD AA 6–38 deletion

**Fig 5:**
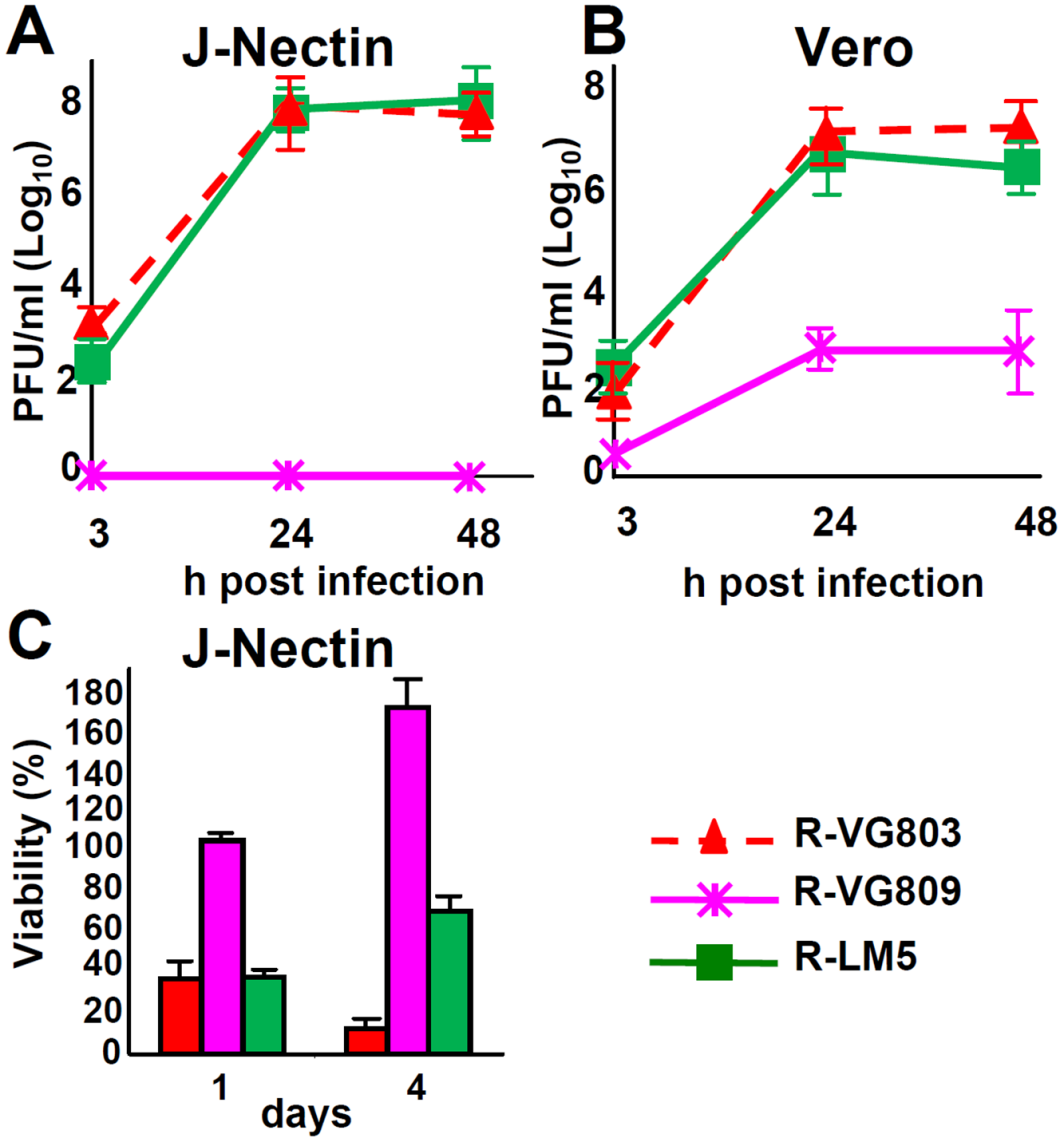
Detargeting conferred to R-VG809 by the AA 6–38 deletion in gD. (A, B) Growth curves of R-VG803, R-VG809 and R-LM5 in J-nectin (A) and Vero (B) cells, infected at an input multiplicity of infection of 0.1 or 1 PFU/cell, respectively, and harvested at the indicated times (h) after infection. Progeny viruses were titrated in SK-OV-3 cells. (C) Cell killing ability of R-VG803, R-VG809 and R-LM5 for J-nectin cells. Cells were infected with the indicated viruses at 2 PFU/cell. Cell viability was determined by AlamarBlue, in triplicate monolayers, at the indicated days after infection. Each point or column represents the mean of triplicates ± S.D.

We characterized the detargeting effect exerted by the AA 6–38 deletion in gD. The retargeted phenotype exhibited by R-LM113 may result from the deletion *per se* or from the combined deletion-insertion. For example, the scFv insert, which is ~ 270 AA long, is likely to induce distortions in gD N-terminus, such that it can not any longer interact with the core of the molecule. Moreover, the insert may mask part of the nectin1 binding site in gD. To discriminate among these possibilities we measured R-VG809 replication in J-nectin1 and Vero cells. We included R-VG803 and R-LM5 for comparison. Fig 5A and 5B shows that R-VG809, but not R-VG803 and R-LM5, failed to replicate in J-nectin and Vero cells. Also the cytolytic effect of R-VG809 was strikingly different from those of R-VG803 and R-LM5, in that R-VG809 failed to kill J-nectin cells (Fig 5C). Parenthetically, the increase in cells viability exhibited by R-VG809 and R-LM5 between day 1 and 4 may be consequent to fact that some cells were not infected at day 0, and they replicated in the time interval of the assay. We conclude that the of AA 6–38 deletion in gD suffices to achieve full detargeting from both HVEM and nectin1, even in the absence of any insert.

### Replication and cell killing ability of R-VG803 and R-VG809

**Fig 6:**
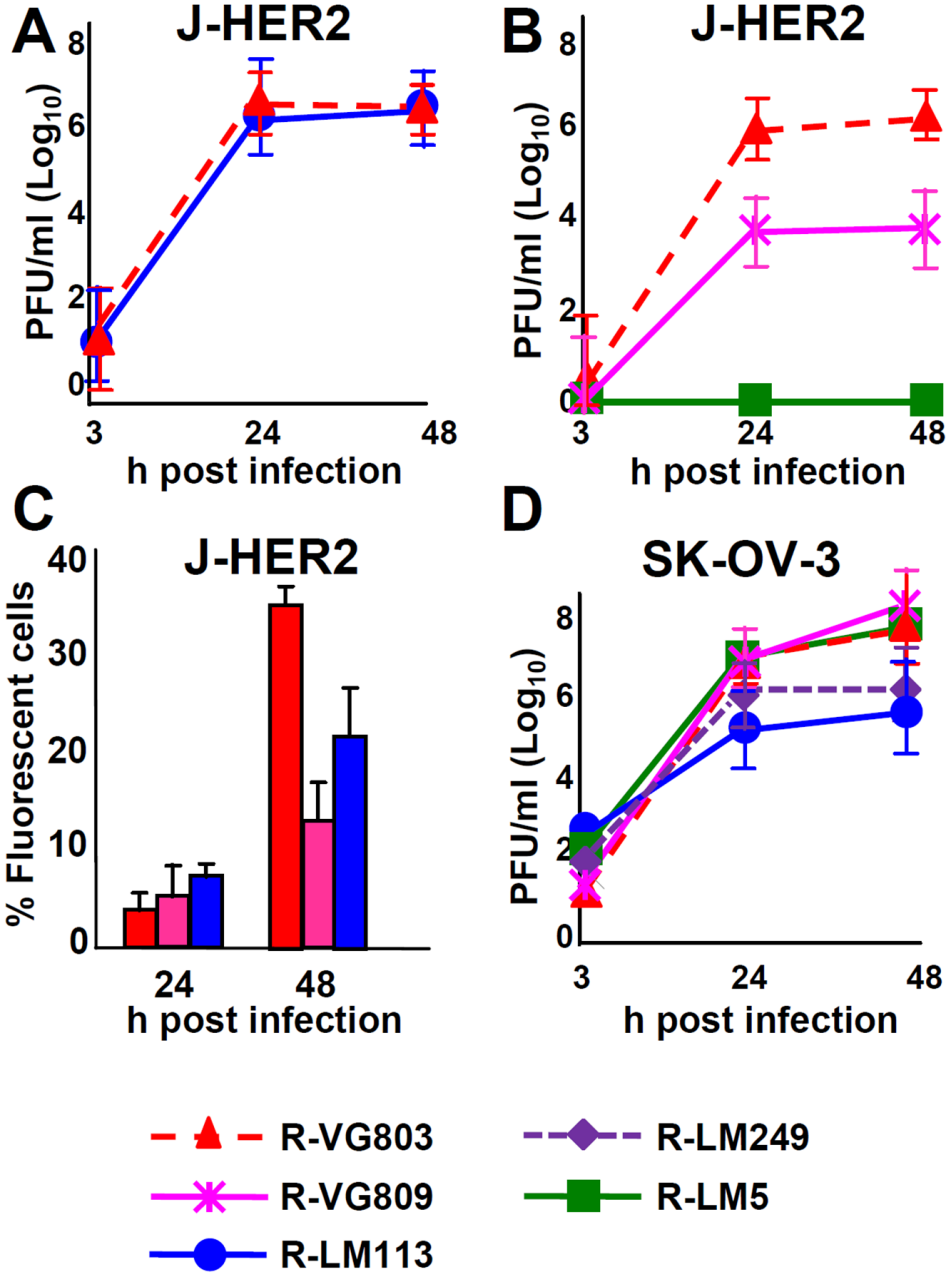
Replication of R-VG803 and R-VG809 in J-HER2 and SK-OV-3 cells. (A, B) Growth curves of R-VG803, R-VG809, R-LM113, R-LM5 in J-HER2. J-HER-2 cells were infected at 0.1 PFU/cell (A), or 0.01 PFU/cell (B). The viral titres of the input viruses were determined in the same cell line. Progeny virus was harvested at the indicated times and titrated in J-HER2 cells. (C) Comparison of cell-to-cell spread by R-VG803, R-VG809 and R-LM113 in J-HER2 cells. J-HER2 cell monolayers were infected with the indicated viruses at 0.01 PFU/cell. The percentage of fluorescent cells at 24 and 48 h after infection was determined by flow cytometry. (D) Growth curves of the indicated viruses (0.1 PFU/cell) in SK-OV-3. Input and progeny viruses were titrated in SK-OV-3 cells. Results are the average of at least two independent experiments ± S.D.

Replication efficiency and cell killing are key properties for any candidate o-HSV. We verified the replication efficiency of R-VG803 and R-VG809 in J-HER2 cells, in comparison to that of R-LM113, R-LM249 and R-LM5. Fig 6A shows that the yields of R-VG803 and R-LM113 in cells infected at 0.1 PFU/cell were practically undistinguishable, implying that the extent of replication in J-HER2 cells is independent of whether the retargeting is achieved through gH or gD. Fig 6B compares the yields of R-VG803, R-VG809 and R-LM5 in J-HER2 cells infected at 0.01 PFU/cell. R-VG809 was somewhat hampered relative to R-VG803. R-VG809 was capable of cell-to-cell spread in J-HER2 cells; the decrease relative to R-VG803 likely reflected the lower extent of replication than the spread *per se* (Fig 6C).

Of interest was the growth in SK-OV-3 cells, as these are cancer cells, resistant to trastuzumab [35]. R-VG803 and R-VG809 replicated equally well, could not be differentiated from the wt R-LM5, and replicated somewhat better than R-LM113 and R-LM249 (Fig 6D).

**Fig 7:**
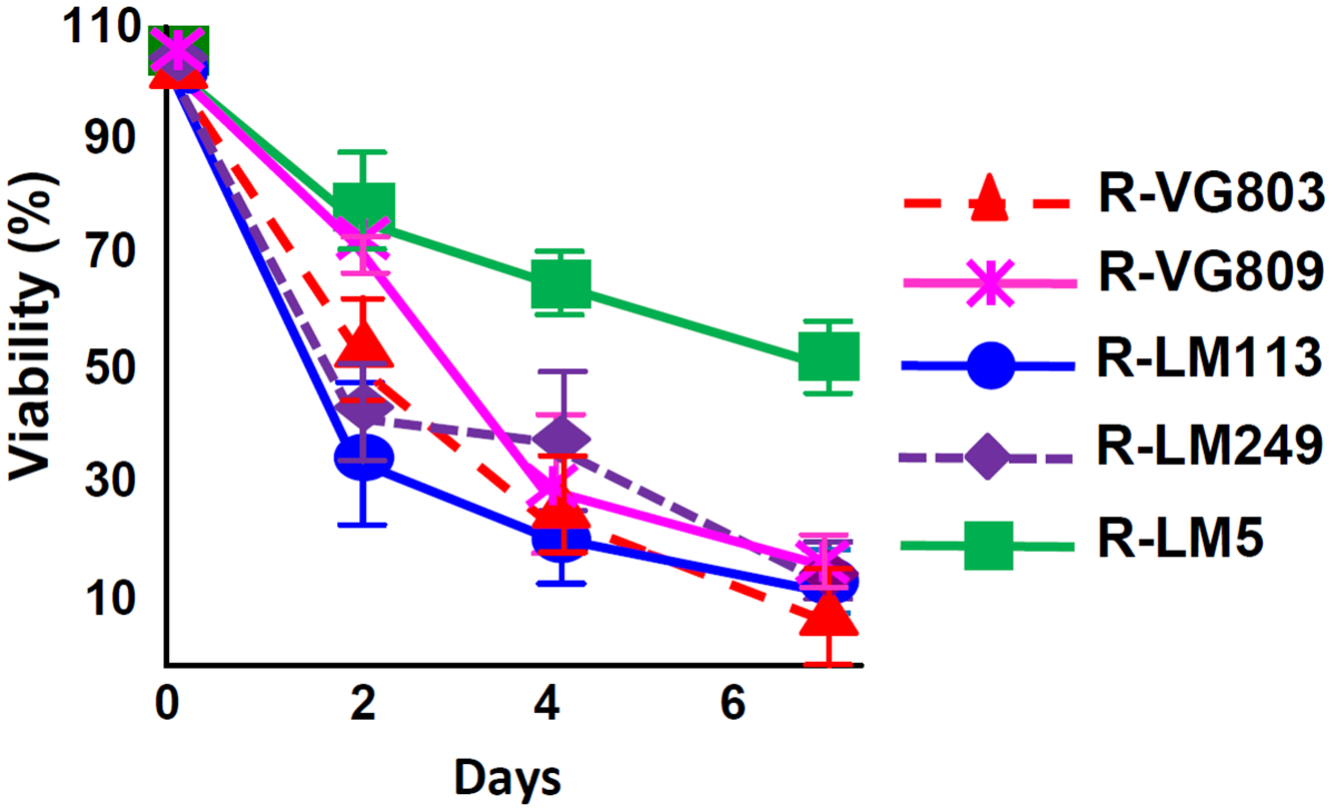
Cell killing ability of R-VG803 and R-VG809 for SK-OV-3 cells. Cells were infected with the indicated viruses at 2 PFU/cell. Cell viability was determined by AlamarBlue, in triplicate monolayers. Each figure represents the average of triplicates ± S.D.

Lastly, we analyzed the ability of R-VG803, R-VG809 to kill the SK-OV-3 tumor cells, in comparison to R-LM113, R-LM249 and R-LM5. Cytotoxicity caused by R-VG803, R-VG809, R-LM113 and R-LM249 were very similar one to the other, and much higher than that of R-LM5 (Fig 7).

## Discussion

The engineering of a novel ligand—a single chain antibody (scFv) directed to HER2—in gH conferred to HSV an expanded tropism for cells which express HER2 as the sole receptor. Virus entry mediated by the chimeric scFv-HER2–gH could occur in the absence of gD receptors, despite deletion of the receptor-binding sites in gD, or presence of gD-neutralizing MAbs. Basically, the key functions of gD are no longer essential, and can be replaced by a ligand in gH. This finding impacts on current view of how HSV enters cells, and on the strategies for retargeting the HSV tropism to receptors of choice.

When wt-HSV enters target cells, gD serves two major functions. It serves as major receptor binding glycoprotein, and determinant of the viral tropism, i.e. it dictates which cells HSV will or will not infect. Secondly, the encounter of HSV with a cell carrying a gD receptor is signaled to gH/gL and gB, to trigger fusion. In essence, the receptor-bound gD initiates the cascade of activation of the entry glycoproteins [1–3,47,48] The control exerted by gD on virion-cell fusion ensures that the activation of the viral fusion machinery occurs only when HSV has reached a receptor-positive cell. In contrast to wt-virus, when the gH-retargeted viruses infect J-HER2 cells the activation of the chimeric scFv—gH does not require gD activation by its receptor, or receptor-binding sites in gD. gD is functionally ablated as receptor-binding glycoprotein and as activator of the downstream glycoproteins. gD is no longer a requirement to trigger fusion. Its functions have been taken over by the scFv in gH.

In wt virus, the activation exerted by the receptor-bound gD on gH/gL necessarily occurs through intermolecular signaling. We refer to it as *trans*-signaling, as opposed to a signaling that occurs intramolecularly, herein referred to as *cis*-signaling. A novelty of our results is that the activation of gH can occur *in cis*, i.e. the scFv activates the gH moiety in the chimera. In the past, Klupp and Mettenleiter generated a non-viable PrV recombinant, carrying a deletion in gL [49]. Upon serial blind passages, a viable recombinant carrying a gD-gH fusion was isolated [50]. Subsequently, Cairns et al. constructed a similar HSV gD-gH chimera, in which the entire ectodomain of gD was fused to the N-terminus of gH (named chimera 22 in their work) [51]. In complementation assays, the HSV chimera rescued the infection of a gD^-/-^ gH^+^ virus, or of a gH^-/-^ gD^+^ virus. It was not tested for complementation of a double deletion gD^-/-^ gH^-/-^ virus. There are two key differences between the previous report [51] and our finding. First, in the complementation assays, the wt-gD in the gH^-/-^ gD^+^ virus had the possibility to activate *in trans* the gH moiety in the gD-gH chimera. Conversely, the gD moiety in the chimera had the possibility to activate *in trans* the wt-gH present in the gD^-/-^ gH^+^ virions. In either case, the activation may have taken place *in-trans*, as concluded by the Authors. Formal evidence for *cis*-activation of the gD-gH chimera was not provided [51]. Secondly, irrespective of the activation mechanism, in the complementing system the gH activation was mediated by gD, which has a binding site on gH [14–16,52], and not by a heterologous ligand. The latter was indeed an unexpected possibility. Previous attempts to develop systems for HSV-mediated cell fusion, or HSV infection independent of gD led to partial indications as follows. In the cell-cell fusion assay, a partial deletion in the N-terminus of gH was reported to induce low, constitutive levels of fusion by gB, in the absence of gD or gD receptors [48]. Whether, once present in the virion, the same deletion will lead to a constitutive, low level gD-independent entry, or will lead to an exhausted fusion/entry machinery has not been ascertained. Uchida and collaborators reported on mutations in virion gB, or virion gH, that render these glycoproteins independent of gD activation by its major receptor nectin1, but are still dependent on the activation by so-called unconventional gD receptors (e.g. nectin3 present in J cells [14]), or receptors to a retargeted gD (e.g. EGFR for a EGFR-retargeted gD). The ability of the mutant forms of gB or gH to carry out entry independently of any form of gD activation, or with a form of gD deleted in receptor-binding sites was not established [53]. Hence, previous studies are strikingly different from current study, where the receptor-binding activity of gD for nectin1/HVEM was ablated by deletion of key residues, and a heterologous receptor-binding activity was implanted in gH.

As regards the field of oncolytic HSVs, our data show that gH accepted the insertion of a hetelogous ligand and became a tool for the retargeting of HSV tropism to a heterologous receptor. The ligand may be at least 270 AA in size, i.e. about 1/3 of gH ectodomain. The gH-mediated retargeting could be combined with detargeting, through a suitable deletion in gD. This ensued in the fully retargeted R-VG809, whose replication and killing capacity for SK-OV-3 cells did not substantially differ, or were even better than those of the gD-retargeted R-LM113 and R-LM249. In essence, changes in tropism through modifications in gH or in gD yield o-HSVs with substantially similar growth and lytic properties. Remarkably, both the gH- and the gD-retargeted o-HSVs grew almost as efficiently as the wt R-LM5. They represent an improvement over the first generation retargeted o-HSVs, which were marred by a relatively low replication capacity [30,54]. We highlight that so far, gD was the only glycoprotein that successfully enabled the retargeting of HSV [30,33–38,54]. Earlier efforts to use glycoproteins other than gD, e.g. gC, did not meet with success [55].

Current findings expand the toolkit for generation of non attenuated retargeted o-HSVs. Two prospective applications are worth noting. The anti-HER2 huMAbs and small molecule inhibitors of HER2 signaling now in clinical trials have non-overlapping mechanisms of action, and patients clearly benefit from combinations [56,57]. However, a fraction of patients does not respond. The responders develop resistance, often within a year of treatment [58]. In the resistant cancer cells, the HER2 ectodomain is preserved, and the modifications affect the signaling portions of the receptor. This type of resistance is recapitulated in SK-OV-3 cells, which are HER2^+^ and trastuzumab-resistant [35]. The observation that R-VG809, as well as R-LM249 [34,35], can grow and kill SK-OV-3 cells raises the possibility that treatment with HER2-retargeted o-HSVs could be applied to patients who developed resistance to the anti-HER2 specific therapeutics. Secondly, the heterogeneity in cancers cells represents a limit to numerous therapeutic approaches. Heterogeneity is observed also in the extent of expression of cancer receptors. The possibility to retarget o-HSV tropism to cancer receptors *via* gH and *via* gD opens the way to the design of double-retargeted o-HSVs, which may be better suited to counteract cancer cell heterogeneity than singly-retargeted o-HSVs.

## Materials and Methods

### Cells and viruses

The receptor negative J cells, their counterparts expressing HER2, nectin1, HVEM and CHO-HER2 were described [7,43]. HFF14 cells were received by Dr. Frank Neipel (University of Erlangen). Vero, RS, SK-OV-3, HaCaT, BHK, HeLa and SK-N-SH cells were received by ATCC. The wt- HSV-1(F), R-LM113, R-LM249 and R-LM5 were described [33,34,43,59].

### Engineering of R-VG803 and R-VG809

First, we engineered R-VG801, by insertion of the sequence encoding the trastuzumab scFv between AA 23 and 24 of gH. Subsequently we engineered R-VG803 by insertion of mCherry sequences into the UL37-UL38 intergenic region of R-VG801. To generate R-VG801, the starting viral genome was pYEBac102, which carries LOX-P-bracketed p-BeloBAC sequences inserted between UL3 and UL4 of HSV-1 genome [60]. All engineering procedures were performed by means of galK recombineering [61]. Briefly, the GalK cassette, with homology arms to gH was amplified by means of primers gH6_galK_f ATGCGGTCCATGCCCAGGCCATCCAAAAACCATGGGTCTGTCTGCTCAGTCCTGTTGACAATTAATCATCGGCA and gH5_galK_r TCGTGGGGGTTATTATTTTGGGCGTTGCGTGGGGTCAGGTCCACGACTGGTCAGCACTGTCCTGCTCCTT. This cassette was electroporated in SW102 bacteria carrying pYEBac102. The recombinant clones carrying the galK cassette were selected on M63 plates (15 mM (NH_4_)_2_SO_4_, 100 mM KH_2_PO_4_, 1.8 μg FeSO_4_·7H_2_O, adjusted to pH 7) supplemented with 1 mg/L D-biotin, 0.2% galactose, 45 mg/L L-leucine, 1 mM MgSO_4_·7H_2_O and 12 μg/ml chloramphenicol. To exclude galK false positive colonies, the recombinant clones were plated on McConkey agar base plates, supplemented with 1% galactose and 12 μg/ml chloramphenicol, and checked by colony PCR with primer galK_129_f ACAATCTCTGTTTGCCAACGCATTTGG and galK_417_r CATTGCCGCTGATCACCATGTCCACGC. Next, the trastuzumab scFv cassette, bracketed by Ser-Gly linkers and by upstream and downstream homology arms to gH, was amplified using pSG-ScFvHER2-SG (a gift from Alfredo Nicosia) as template. pSG-ScFvHER2-SG was obtained by inserting the synthetic antiHER2 scFv cassette, designed on the basis of published information [62]; Sequence 18 from Patent WO2004065416 (Genbank CQ877234); Sequence 7 (pS2072a) from Patent WO2005100399 (Genbank CS276173) into an appropriate vector. The scFv cassette was bracketed by the Ser-Gly linkers detailed below. Relative to sequence 18 from Patent WO2004065416, nucleotides 769–771 were mutated in pSG-ScFvHER2-SG to generate a XhoI restriction site. Using pSG-ScFvHER2-SG as template, two separate fragments (# 1 and # 2) were PCR-amplified by means of oligonucleotides which contained homology arms to gH. Specifically, fragment # 1 was amplified by means of primers gH23_8SG_scFv4D5_f TCGTGGGGGTTATTATTTTGGGCGTTGCGTGGGGTCAGG TCCACGACTGGCATAGTAGTGGCGGTGGCTCTGGATCCG and scFv4D5_358_r GGAAACGGTTCGGATCAGCCATCGG, using pSG-ScFvHER2-SG as template. Fragment # 2 was amplified by means of gH24_12SG_scFv4D5r ATGCGGTCCATGCCCAGGCCATCCAAAAACCATGGGTCTGTCTGCTCAGTACCG GATCCACCGGAACCAGAGCC and scFv4D5_315_f GGAGATCAAATCGGATATGCCGATGG using pSG-ScFvHER2-SG as template. Thereafter, fragments # 1 and # 2 were annealed and extended to generate the entire scFv-HER2 cassette, bracketed by the Ser-Gly linkers and the homology arms to gH. The sequence of the upstream and downstream Ser-Gly linkers were HSSGGGSG, and SSGGGSGSGGSG, respectively. The linker between V_L_ and V_H_ had the sequence SDMPMADPNRFRGKNLVFHS. The recombinant bacterial clones carried the scFv-HER2 cassette in place of the galK cassette. They were selected on M63 plates, supplemented with 1 mg/L D-biotin, 0.2% deoxy-2-galactose, 0.2% glycerol, 45 mg/L L-leucine, 1 mM MgSO_4_·7H_2_O and 12 μg/ml chloramphenicol. Bacterial colonies were checked for the presence of inserted sequence by colony PCR.

The mCherry red fluorescent protein, under the CMV promoter, was inserted in the UL37-UL38 intergenic region of R-VG801 (coordinates 84156–84157), to generate R-VG803, following the two step procedure outlined above. Briefly, we first inserted the galK cassette, amplified by means of oligonucleotides UL37/38_galK_f CCGCAGGCGTTGCGAGTACCCCGCGTCTTCGCGGGGTGTTATACGGCCACCCTGTTGACAATTAATCATCGGCA and UL37/38_galK_r TCCGGACAATCCCCCGGGCCTGGGTCCGCGAACGGGATGCCGGGACTTAATCAGCACTGTCCTGCTCCTT. Subsequently, the galK sequence was replaced with the promoter-mCherry cassette, amplified by means of oligonucleotides UL37/38_CMV_mcherry_f CCGCAGGCGTTGCGAGTACCCCGCGTCTTCGCGGGGTGTTATACGGCCACCGATGTACGGGCCAGATATACG and UL37/38_pA_mcherry_1958_r TCCGGACAATCCCCCGGGCCTGGGTCCGCGAACGGGATGCCGGGACTTAACCATAGAGCCCACCGCATCC. The starting material for R-VG809 was the R-VG803 BAC genome. To generate the AA 6–38 deletion in gD, a galK cassette flanked by homology arms to gD was amplified by means of primers gD5_galK_f TTGTCGTCATAGTGGGCCTCCATGGGGTCCGCGGCAAATATGCCTTGGCGCCTGTTGACAATTAATCATCGGCA and gD39_galK_r ATCGGGAGGCTGGGGGGCTGGAACGGGTCCGGTAGGCCCGCCTGGATGTGTCAGCACTGTCCTGCTCCTT. Next, we replaced the galK sequence with a synthetic double stranded oligonucleotide gD_aa5_39_f_r TTGTCGTCATAGTGGGCCTCCATGGGGTCCGCGGCAAATATGCCTTGGCGCACATCCAGGCGGGCCTACCGGACCCGTTCCAGCCCCCCAGCCTCCCGAT. In all cases, the recombinant viruses were generated by transfection of SK-OV-3 cells with the appropriate recombinant BAC DNA (500 ng) by means of Lipofectamine 2000 (Life Technologies). Virus growth was monitored by red fluorescence. The structure of the viral recombinants was verified by sequencing the gH and mCherry ORFs, and gD ORF for R-VG809. Virus stocks were generated and titrated in SK-OV-3 cells, or in J-HER2 cells, as specified.

### Expression of chimeric gH from R-VG803 and R-VG809

Lysates of Vero cells infected with R-VG803, R-VG809 or R-LM5 (3 PFU/cell) were subjected to PAGE, transferred to PVDF membranes. Immunoblot reactivity to polyclonal antibody (PAb) to gH was assayed as detailed [44].

### Tropism of R-VG803 and R-VG809

The indicated cells were infected with R-VG803, or R-VG809 at 2 and 20 PFU/cell, respectively. Red fluorescence was monitored by fluorescence microscopy.

### Block of R-VG803 and R-VG809 infection by MAbs to HER2 and gD

Replicate monolayers of J-HER2 or SK-OV-3 cells in 12 well plates were preincubated with trastuzumab or non-immune mouse IgG (28 μg/ml, final concentration) for 1 h, and then infected with R-VG803, R-VG809, R-LM113, R-LM249 or R-LM5 (0.3 or 2 PFU/cell for J-HER2 or SK-OV-3 cells, respectively), in the same medium. Alternatively, virions were preincubated with MAbs HD1 (1.5 μg/ml, final concentration), or MAb 52 S (ascites fluid 1:25) for 1 h at 37°C, and then allowed to absorb to cells for 90 min, in the absence or presence of trastuzumab, as indicated. Viral inocula were then removed, and cells were overlaid with medium containing the indicated antibodies. Virus replication was monitored at 24 h after infection by BD Accuri C6 flow cytometer. Results are expressed ad the mean of three independent experiments ± SD.

### Virus growth determinations

To determine R-VG803 and R-VG809 growth in J-HER2, SK-OV-3, J-nectin, and Vero cells, the cells were infected with R-VG803, R-LM113, R-LM249, R-LM5 at the indicated MOI. Unabsorbed virus was inactivated by rinsing cells with pH 3 solution (40 mM citric acid, 10 mM KCl, 135 mM NaCl). Cells were harvested at 3 (0 time), 24 and 48 h after infection and progeny virus (intracellular plus extracellular) was titrated in J-HER2 or SK-OV-3 cells, as indicated.

### Cytotoxity assay

SK-OV-3 and J-nectin cells were seeded in 96 well plates at 8x10^3^ cell/well, and infected with R-VG803, R-VG809, R-LM113, R-LM249 and R-LM5 (2 PFU/cell) or mock-infected. AlamarBlue (10 μl/well, Life Technologies) was added to the culture media at indicated times after infection and incubated for 4 h at 37°C. Plates were read at 560 and 600 nm with GloMax Discover System (Promega). For each time point, cell viability was expressed as the percentage of AlamarBlue reduction in infected *versus* uninfected cells, excluding for each set of samples the contribution of medium alone. Each point represents the average of at least triplicate samples ± SD.

### Accession numbers

HSV-1 strain F, Genbank GU73477. gH coordinates 43783–46299

HSV-1 strain F, gD: Genbank L09242

scFv to HER2 Genbank CQ877234, CS276173

mCherry Genbank HM771696

## Supporting Information

S1 Fig Annotated sequence of gH-scFv chimera in R-VG803 and R-VG809. Regions of interest are highlighted as follows. Signal sequence, yellow. scFv to HER2, blue. Upstream and downstream Ser-Gly linkers, green. scFv intermediate linker, red. Two residues implicated in MAb 52S epitope, S536 and A537 in wt-gH, fuchsia.
(PDF)
