# Supplementary material for: The Engineering of a Novel Ligand in gH Confers to HSV an Expanded Tropism Independent of gD Activation by Its Receptors
Source: PLoS Pathog. 2015 May 21;11(5):e1004907. doi: 10.1371/journal.ppat.1004907 (PMC4440635; doi:10.1371/journal.ppat.1004907)
Supplement: S1 Fig — Regions of interest are highlighted as follows. Signal sequence, yellow. scFv to HER2, blue. Upstream and downstream Ser-Gly linkers, green. scFv intermediate linker, red. Two residues implicated in MAb 52S epitope, S536 and A537 in wt-gH, fuchsia. (PDF) [file ppat.1004907.s001.pdf]

SUPPORTING INFORMATION

S1 Fig.

```
   1 Met Gly Asn Gly Leu Trp Phe Val Gly Val Ile Ile Leu Gly Val Ala Trp Gly Gln Val   20
  21 His Asp Trp His Ser Ser Gly Gly Gly Ser Gly Ser Asp Ile Gln Met Thr Gln Ser Pro   40
  41 Ser Ser Leu Ser Ala Ser Val Gly Asp Arg Val Thr Ile Thr Cys Arg Ala Ser Gln Asp   60
  61 Val Asn Thr Ala Val Ala Trp Tyr Gln Gln Lys Pro Gly Lys Ala Pro Lys Leu Leu Ile   80
  81 Tyr Ser Ala Ser Phe Leu Tyr Ser Gly Val Pro Ser Arg Phe Ser Gly Ser Arg Ser Gly  100
 101 Thr Asp Phe Thr Leu Thr Ile Ser Ser Leu Gln Pro Glu Asp Phe Ala Thr Tyr Tyr Cys  120
 121 Gln Gln His Tyr Thr Thr Pro Pro Thr Phe Gly Gln Gly Thr Lys Val Glu Ile Lys Ser  140
 141 Asp Met Pro Met Ala Asp Pro Asn Arg Phe Arg Gly Lys Asn Leu Val Phe His Ser Glu  160
 161 Val Gln Leu Val Glu Ser Gly Gly Gly Leu Val Gln Pro Gly Gly Ser Leu Arg Leu Ser  180
 181 Cys Ala Ala Ser Gly Phe Asn Ile Lys Asp Thr Tyr Ile His Trp Val Arg Gln Ala Pro  200
 201 Gly Lys Gly Leu Glu Trp Val Ala Arg Ile Tyr Pro Thr Asn Gly Tyr Thr Arg Tyr Ala  220
 221 Asp Ser Val Lys Gly Arg Phe Thr Ile Ser Ala Asp Thr Ser Lys Asn Thr Ala Tyr Leu  240
 241 Gln Met Asn Ser Leu Arg Ala Glu Asp Thr Ala Val Tyr Tyr Cys Ser Arg Trp Gly Gly  260
 261 Asp Gly Phe Tyr Ala Met Asp Tyr Trp Gly Gln Gly Thr Leu Val Thr Val Ser Ser Ser  280
 281 Gly Gly Gly Ser Gly Ser Gly Gly Ser Gly Thr Glu Gln Thr Asp Pro Trp Phe Leu Asp  300
 301 Gly Leu Gly Met Asp Arg Met Tyr Trp Arg Asp Thr Asn Thr Gly Arg Leu Trp Leu Pro  320
 321 Asn Thr Pro Asp Pro Gln Lys Pro Pro Arg Gly Phe Leu Ala Pro Pro Asp Glu Leu Asn  340
 341 Leu Thr Thr Ala Ser Leu Pro Leu Leu Arg Trp Tyr Glu Glu Arg Phe Cys Phe Val Leu  360
 361 Val Thr Thr Ala Glu Phe Pro Arg Asp Pro Gly Gln Leu Leu Tyr Ile Pro Lys Thr Tyr  380
 381 Leu Leu Gly Arg Pro Pro Asn Ala Ser Leu Pro Ala Pro Thr Thr Val Glu Pro Thr Ala  400
 401 Gln Pro Pro Pro Ser Val Ala Pro Leu Lys Gly Leu Leu Tyr Asn Pro Val Ala Ser Val  420
 421 Leu Leu Arg Ser Arg Ala Trp Val Thr Phe Ser Ala Val Pro Asp Pro Glu Ala Leu Thr  440
 441 Phe Pro Arg Gly Asp Asn Val Ala Thr Ala Ser His Pro Ser Gly Pro Arg Asp Thr Pro  460
 461 Pro Pro Arg Pro Pro Val Gly Ala Arg Arg His Pro Thr Thr Glu Leu Asp Ile Thr His  480
 481 Leu His Asn Ala Ser Thr Thr Trp Leu Ala Thr Arg Gly Leu Leu Arg Ser Pro Gly Arg  500
 501 Tyr Val Tyr Phe Ser Pro Ser Ala Ser Thr Trp Pro Val Gly Ile Trp Thr Thr Gly Glu  520
 521 Leu Val Leu Gly Cys Asp Ala Ala Leu Val Arg Ala Arg Tyr Gly Arg Glu Phe Met Gly  540
 541 Leu Val Ile Ser Met His Asp Ser Pro Pro Val Glu Val Met Val Val Pro Ala Gly Gln  560
 561 Thr Leu Asp Arg Val Gly Asp Pro Ala Asp Glu Asn Pro Pro Gly Ala Leu Pro Gly Pro  580
 581 Pro Gly Gly Pro Arg Tyr Arg Val Phe Val Leu Gly Ser Leu Thr Arg Ala Asp Asn Gly  600
 601 Ser Ala Leu Asp Ala Leu Arg Arg Val Gly Gly Tyr Pro Glu Glu Gly Thr Asn Tyr Ala  620
 621 Gln Phe Leu Ser Arg Ala Tyr Ala Glu Phe Phe Ser Gly Asp Ala Gly Ala Glu Gln Gly  640
 641 Pro Arg Pro Pro Leu Phe Trp Arg Leu Thr Gly Leu Leu Ala Thr Ser Gly Phe Ala Phe  660
 661 Val Asn Ala Ala His Ala Asn Gly Ala Val Cys Leu Ser Asp Leu Leu Gly Phe Leu Ala  680
 681 His Ser Arg Ala Leu Ala Gly Leu Ala Ala Arg Gly Ala Ala Gly Cys Ala Ala Asp Ser  700
 701 Val Phe Phe Asn Val Ser Val Leu Asp Pro Thr Ala Arg Leu Gln Leu Glu Ala Arg Leu  720
 721 Gln His Leu Val Ala Glu Ile Leu Glu Arg Glu Gln Ser Leu Ala Leu His Ala Leu Gly  740
 741 Tyr Gln Leu Ala Phe Val Leu Asp Ser Pro Ser Ala Tyr Asp Ala Val Ala Pro Ser Ala  760
 761 Ala His Leu Ile Asp Ala Leu Tyr Ala Glu Phe Leu Gly Gly Arg Val Leu Thr Thr Pro  780
 781 Val Val His Arg Ala Leu Phe Tyr Ala Ser Ala Val Leu Arg Gln Pro Phe Leu Ala Gly  800
 801 Val Pro Ser Ala Val Gln Arg Glu Arg Ala Arg Arg Ser Leu Leu Ile Ala Ser Ala Leu  820
 821 Cys Thr Ser Asp Val Ala Ala Ala Thr Asn Ala Asp Leu Arg Thr Ala Leu Ala Arg Ala  840
 841 Asp His Gln Lys Thr Leu Phe Trp Leu Pro Asp His Phe Ser Pro Cys Ala Ala Ser Leu  860
 861 Arg Phe Asp Leu Asp Glu Ser Val Phe Ile Leu Asp Ala Leu Ala Gln Ala Thr Arg Ser  880
 881 Glu Thr Pro Val Glu Val Leu Ala Gln Gln Thr His Gly Leu Ala Ser Thr Leu Thr Arg  900
 901 Trp Ala His Tyr Asn Ala Leu Ile Arg Ala Phe Val Pro Glu Ala Ser His Arg Cys Gly  920
 921 Gly Gln Ser Ala Asn Val Glu Pro Arg Ile Leu Val Pro Ile Thr His Asn Ala Ser Tyr  940
 941 Val Val Thr His Ser Pro Leu Pro Arg Gly Ile Gly Tyr Lys Leu Thr Gly Val Asp Val  960
 961 Arg Arg Pro Leu Phe Leu Thr Tyr Leu Thr Ala Thr Cys Glu Gly Ser Thr Arg Asp Ile  980
 981 Glu Ser Lys Arg Leu Val Arg Thr Gln Asn Gln Arg Asp Leu Gly Leu Val Gly Ala Val 1000
1001 Phe Met Arg Tyr Thr Pro Ala Gly Glu Val Met Ser Val Leu Leu Val Asp Thr Asp Asn 1020
1021 Thr Gln Gln Gln Ile Ala Ala Gly Pro Thr Glu Gly Ala Pro Ser Val Phe Ser Ser Asp 1040
1041 Val Pro Ser Thr Ala Leu Leu Leu Phe Pro Asn Gly Thr Val Ile His Leu Leu Ala Phe 1060
1061 Asp Thr Gln Pro Val Ala Ala Ile Ala Pro Gly Phe Leu Ala Ala Ser Ala Leu Gly Val 1080
1081 Val Met Ile Thr Ala Ala Leu Ala Gly Ile Leu Lys Val Leu Arg Thr Ser Val Pro Phe 1100
1101 Phe Trp Arg Arg Glu End  1106
```

Annotated sequence of gH-scFv chimera in R-VG803 and R-VG809. Regions of interest are highlighted as follows. Signal sequence, yellow. scFv to HER2, blue. Upstream and downstream Ser-Gly linkers, green. scFv intermediate linker, red. Two residues implicated in MAb 52S epitope, S536 and A537 in wt-gH, fuchsia.
